# Characterization of T2-Low and T2-High Asthma Phenotypes in Real-Life

**DOI:** 10.3390/biomedicines9111684

**Published:** 2021-11-13

**Authors:** Fabio Luigi Massimo Ricciardolo, Andrea Elio Sprio, Andrea Baroso, Fabio Gallo, Elisa Riccardi, Francesca Bertolini, Vitina Carriero, Elisa Arrigo, Giorgio Ciprandi

**Affiliations:** 1 Department of Clinical and Biological Sciences, University of Turin, San Luigi Gonzaga University Hospital, 10043 Turin, Italy; andrea.sprio@unito.it (A.E.S.); andrea.baroso@unito.it (A.B.); elisa.riccardi@unito.it (E.R.); francesca.bertolini@unito.it (F.B.); vitina.carriero@unito.it (V.C.); elisa.arrigo@unito.it (E.A.); 2 Department of Research, ASOMI College of Sciences, 19112 Marsa, Malta; 3 Clinical Epidemiology Unit, IRCCS Ospedale Policlinico San Martino, 16132 Genoa, Italy; fabio9980@gmail.com; 4 Allergy Clinic, Casa di Cura Villa Montallegro, 16145 Genoa, Italy; gio.cip@libero.it

**Keywords:** asthma, inflammation, asthma phenotypes, real-world, clinical practice, T2 low, T2 high

## Abstract

Asthma is a heterogeneous and complex condition characterized by chronic airway inflammation, which may be clinically stratified into three main phenotypes: type 2 (T2) low, T2-high allergic, and T2-high non-allergic asthma. This real-world study investigated whether phenotyping patients with asthma using non-invasive parameters could be feasible to characterize the T2-low and T2-high asthma phenotypes in clinical practice. This cross-sectional observational study involved asthmatic outpatients (*n* = 503) referring to the Severe Asthma Centre of the San Luigi Gonzaga University Hospital. Participants were stratified according to the patterns of T2 inflammation and atopic sensitization. Among outpatients, 98 (19.5%) patients had T2-low asthma, 127 (25.2%) T2-high non-allergic, and 278 (55.3%) had T2-high allergic phenotype. In comparison to T2-low, allergic patients were younger (OR 0.945, *p* < 0.001) and thinner (OR 0.913, *p* < 0.001), had lower smoke exposure (OR 0.975, *p* < 0.001) and RV/TLC% (OR 0.950, *p* < 0.001), higher prevalence of asthma severity grade 5 (OR 2.236, *p* < 0.05), more frequent rhinitis (OR 3.491, *p* < 0.001) and chronic rhinosinusitis with (OR 2.650, *p* < 0.001) or without (OR 1.919, *p* < 0.05) nasal polyps, but less common arterial hypertension (OR 0.331, *p* < 0.001). T2-high non-allergic patients had intermediate characteristics. Non-invasive phenotyping of asthmatic patients is possible in clinical practice. Identifying characteristics in the three main asthma phenotypes could pave the way for further investigations on useful biomarkers for precision medicine.

## 1. Introduction

Asthma is a heterogeneous and complex disorder characterized by different phenotypes and endotypes. Airway inflammation is the pathophysiologic mainstay [1] caused by different pathogenic mechanisms. Thereby, a recent approach tends to identify the underlying immune-pathological characteristics. In this regard, three kinds of cell-mediated immune responses (type 1, type 2, and type 3) can be highlighted based on the presence of specific lineages of effector T-cell and innate lymphoid cells (ILC) [2]. Type 1 immunity comprises interferon (IFN)-γ and tumor necrosis factor (TNF) producing T_H_1, T_C_1, and ILC1 cells and mainly focuses against intracellular microbes; type 2 effectors (T_H_2, T_C_2, and ILC2) secrete interleukin (IL)-4, IL-5, and IL-13 and have been linked with protection against parasites, allergens, and irritants; type 3 immune response is based on IL-17 producing cells (T_H_17, T_C_17, and ILC3) and historically targets extracellular microbes like bacteria and fungi [2]. Beyond these activities, the three types of immunity concur to the asthma heterogeneity and affect the composition of granulocytic airway infiltrate, leading to four inflammatory phenotypes: eosinophilic, neutrophilic, pauci-granulocytic, and mixed-granulocytic asthma.

Type 2 (T2) inflammation is predominant in asthma and is characterized by eosinophilic airway infiltrate and T_H_2-dependent cytokine overexpression (IL-4, IL-5, and IL-13) [3]. IL-5 mediates differentiation, activation, and survival of eosinophils, while IL-4/13 is essential to induce B cells to produce IgE. This so-called T2-high asthma includes the allergic and the non-allergic phenotype [4,5]. In the allergic phenotype, IL-5 and IL-13 are produced by T_H_2 upon contact with allergens and by ILC2 in response to allergen-driven production of alarmins (IL-25, IL-33, and thymic stromal lymphopoietin - TSLP) in epithelial cells [6]. This phenotype is characterized by early onset and production of allergen-specific IgE (sensitization). Otherwise, in the non-allergic T2-high phenotype, innate immunity is mediated with non-specific mechanisms [5], as pollutants, parasites and pathogens can trigger airway hyperresponsiveness and eosinophilia through alarmin-mediated ILC2 activation. Clinically, non-allergic eosinophilic patients had late-onset asthma and often developed chronic rhinosinusitis with nasal polyps (CRSwNP) [6].

Neutrophilic and pauci-granulocytic airway infiltrates are enclosed in the T2-low asthma and are promoted by IL-6, IL-8, IL-17, IL-22, and epithelial cell-derived cytokines belonging to type 1 and 3 immunity [6,7]. The T2-low asthma is uncommon in children and adolescents but occurs more frequently in late-onset asthmatics, females, and obese subjects [7]. Further, type 3 inflammation has been associated explicitly with frequent asthma exacerbations [8,9].

Asthma phenotyping allowed us to tailor personalized treatment in asthma management from a conceptual perspective. The most relevant information on this topic derived from big trials, which adopted very selective inclusion and exclusion criteria or investigational international studies that used sophisticated biomarkers [10]. This evidence scarcely mirrors what occurs in clinical practice, thus real-world studies are useful [11]. We tested the hypothesis that managing asthmatics in a real-world setting through non-invasive phenotyping could provide relevant information to characterize the T2-low and T2-high asthma in clinical practice.

## 2. Materials and Methods

### 2.1. Study Design and Patients

The present cross-sectional observational study was approved by the local Ethics Committee (San Luigi Gonzaga University Hospital: protocol 4478/2017, approved on 20 March 2017) and conducted according to the Declaration of Helsinki between June 2017 and December 2020. Study candidates were consecutively recruited among mild-to-severe asthmatics referring to the Severe Asthma Centre of the San Luigi Gonzaga University Hospital. According to the Global Initiative for Asthma (GINA) criteria [12], inclusion criteria were adulthood and documented asthma diagnosis, based on a typical history of respiratory symptoms associated with variable airflow limitation as demonstrated by reversibility to bronchodilators and bronchial hyper-responsiveness to methacholine. Patients with stable asthma [13] discontinued short-acting and long-acting bronchodilators 24 h before lung function measurement. Patients with asthma-COPD overlap (ACO) based on the GINA-GOLD definition [14] were excluded. Smoking habit and history were not assumed per se as an exclusion criterion; patients were considered with history of current or past smoking habits if they were cumulatively exposed to ≥10 pack-year (PY) [15,16].

Patients with poor adherence (<50%) to the treatment or inadequate inhalation technique were excluded to evaluate optimally treated patients solely. Written informed consent was obtained from all subjects.

T2-high inflammation was defined when at least one of the following conditions occurred: blood eosinophils ≥ 300 cells/µL, fractional exhaled nitric oxide (F_E_NO) ≥ 30 ppb, or confirmed allergy [17,18,19]. Total IgE levels were arbitrarily not considered as a T2-high allergic inflammatory biomarker, as it can be elevated also in non-atopic subjects. T2-high patients were further stratified as T2-high allergic or T2-high non-allergic asthma, depending on the presence of confirmed allergy diagnosis [5].

### 2.2. Procedures and Endpoints

Outpatients’ clinical data and history were recorded at recruitment during a follow-up visit. The detailed history included the most common comorbidities. The investigators performed the clinical examination, measured body mass index (BMI), and administered the asthma control test (ACT) questionnaire. The first question of the questionnaire was used to evaluate the activity limitation score.

The use of asthma medications, including inhaled and nasal corticosteroids (CS), theophylline, long-acting beta2-agonists (LABA), long-acting muscarinic antagonists (LAMA), and biologics (omalizumab and mepolizumab) was recorded to assess the treatment step level and define the asthma severity grade after at least six months of follow-up, according to the GINA document [12]. Inhaled corticosteroids (ICS) doses were reported as beclomethasone equivalent. Serum Vitamin D was also titrated, and its supplementation was considered.

The pulmonary function test assessed spirometry and lung volumes using a body plethysmograph (Vmax Encore 62, Carefusion, Würzburg, Germany), as stated by the European Respiratory Society [20]. Bronchodilation testing was performed according to validated criteria [9].

According to the manufacturer’s instructions, F_E_NO was measured using the F_E_NO+ instrument (Medisoft, Sorinnes, Belgium). Exhaled NO was recorded with the single-breath method according to published guidelines [21].

White blood cell (WBC) and WBC differential counts were performed based on optical and impedance characteristics using a Cell-Dyn Sapphire (Abbott, Rome, Italy) automated hematology analyzer.

Allergy was considered if sensitization, such as the production of allergen-specific IgE, was documented by skin prick test and/or serum assay. Moreover, it had to be demonstrated a cause/effect relationship between exposure to the sensitizing allergen and immediate symptom occurrence [17].

For patients already in treatment with biologics, the measurements of F_E_NO and blood eosinophils before the start of therapy were also recovered, as treatment can alter the baseline T2 stratification.

### 2.3. Statistical Analysis

Descriptive statistics of endpoint characteristics were reported as means with standard deviations (SD) for continuous variables and the number of subjects and percentage values for categorical variables. Inflammatory phenotype classification was created, stratifying the patients into two groups: T2-low asthma and T2-high asthma. The latter was further stratified as T2-high non-allergic asthma and T2-high allergic asthma. The analyses were performed between (i) T2-low vs. T2-high as a whole; (ii) T2-low vs. T2-high non-allergic; (iii) T2-low vs. T2-high allergic; (iv) T2-high allergic vs. non-allergic.

The D’Agostino and Pearson test was used to evaluate normality distribution. Outliers were identified with the ROUT method and excluded. Unpaired T-Test or non-parametric Mann–Whitney U-test were used to compare continuous variables, whereas the Fisher F-test was employed to compare categorical variables.

The univariate binomial Logistic Regression (LR) Model screened the T2 phenotype’s clinical and demographic variables. The odds ratios were calculated with their 95% confidence interval as exponentiation of the B-coefficient for each factor from the LR. All detailed results were reported in the Supplementary Table S1. The role of age and smoke exposure as confounding variables for differences in comorbidity prevalence was assessed by a univariate general Linear Regression Model. *p* < 0.05 was set as the significant cut-off. Descriptive statistics were performed with GraphPad Prism version 9.1.1 (GraphPad Software, San Diego, CA, USA), whereas Regression Models were analyzed with IBM SPSS Statistics version 24 (IBM Corp., Armonk, NY, USA). Euler diagram was drawn with “eulerr” package version 6.1.1 for R [22].

## 3. Results

### 3.1. Descriptive Analysis

The present study included 503 (199 males and 304 females) mild-to-severe asthma outpatients whose demographic, biological, physiological, and clinical characteristics are summarized in Table 1.

The mean age was 58.0 years (SD = 15.1), the age at asthma onset was 36.0 years (SD = 18.9), 22.8% of patients had early onset asthma, and the mean BMI was 27.0 (SD = 5.5). Most patients (*n* = 362) had never been smokers, 110 were past smokers, and 31 were current smokers. The mean values of the main lung function parameters were in the normal range. The use of asthma medications is reported in detail. Considering the asthma severity grade, 10.5% patients were in GINA step 1, 11.5% step 2, 29.8% step 3, 22.3% step 4, and 25.8% step 5. The mean ACT value was 20.5 (SD = 3.9); the mean activity limitation score was 4.2 (SD = 1.1).

**Figure 1:**
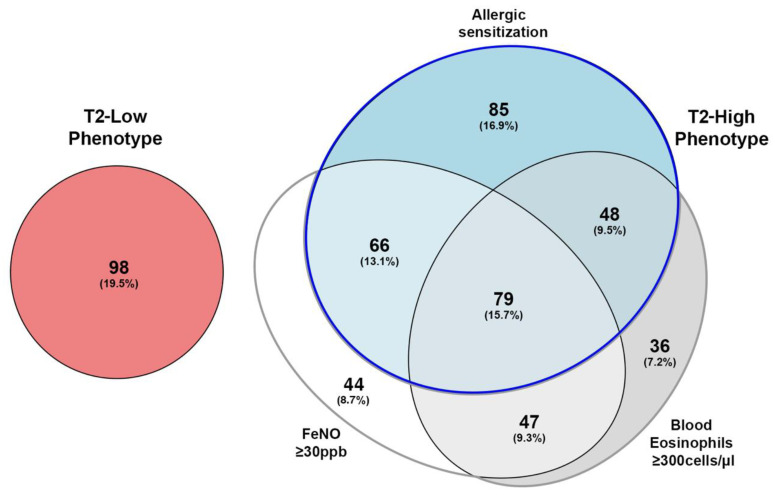
Area-proportional Euler Diagrams of the type 2 biomarker positivity in the type 2 phenotypes, including patients during biologic treatment. The “triple negative” T2-low phenotype is represented in red; in the T2-high phenotype, presence of allergic sensitization is represented in azure; blood eosinophils ≥ 300 cells/µL is reported in grey; F_E_NO ≥ 30 ppb is colored in white; the presence of multiple T2 biomarkers is reported with the overlap of the respective biomarker ovals. The blue line delimits the T2-high allergic phenotype, while the gray line delimits the T2-high non-allergic phenotype.

Figure 1 describes the stratification of patients at the recruitment, considering positivity to each T2 inflammatory biomarker (presence of allergic sensitization, or the exceeding of the threshold value for F_E_NO and blood eosinophils).

Beyond the “triple negative” T2-low phenotype, in which neither allergic sensitization nor the presence of high levels of F_E_NO and blood eosinophils can be detected, seven subgroups could be identified in the T2-high population. Three subgroups were characterized by only one T2 biomarker, while the remaining four showed the simultaneous presence of two or even three T2 biomarkers. As 52 (10.3%) of recruited patients were already in treatment with biologics at recruitment, stratification for asthma phenotypes in our population was performed considering F_E_NO and blood eosinophils measurements either prior (Supplementary Figure S1) or during (Figure 1) the therapy. Changes in T2 phenotype were detected in 21 of 52 patients (40.4%) and reported in Table 2.

Changes accounted mainly for switching between adjacent subgroups inside the T2-high phenotype. Only one patient switched from the “pure eosinophilic” T2-high to the T2-low phenotype following the administration of mepolizumab.

The characterization of T2 phenotypes described below was based on actual biomarkers positivity (Figure 1). Thereby, stratification for asthma phenotypes in our population revealed that 98 (19.5%) patients had T2-low asthma, whereas 405 (80.5%) patients had T2-high asthma, of which 127 (25.2%) showed the T2-high non-allergic phenotype, and 278 (55.3%) the T2-high allergic.

### 3.2. Demographic Characterization of Phenotypes

Demographic characteristics of patients stratified according to T2 asthma phenotypes were reported in Table 3.

**Figure 2:**
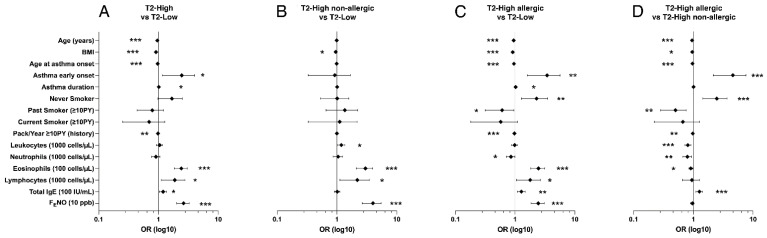
Forest plots of Odds Ratios ± 95% CI concerning demographic characteristics with significant differences between phenotypes at univariate analysis. Type 2 low is considered the reference in panels (**A**–**C**); Type 2 high non-allergic is considered the reference in panel (**D**). * = *p* < 0.05; ** = *p* < 0.01; *** = *p* < 0.001.

As emerged with LR (Figure 2), if compared to the T2-low, subjects with T2-high phenotype were younger (*p* < 0.001, OR = 0.960) and had a lower age at diagnosis (*p* < 0.001, OR = 0.968), therefore were characterized more frequently by early onset (*p* < 0.05, OR = 2.161) and longer asthma duration (*p* < 0.05, OR = 1.016). They were generally thinner (*p* < 0.001, OR = 0.926) and had less exposure to cigarette smoke (PY, *p* < 0.01, OR = 0.984). Finally, T2-high subjects had higher levels of blood eosinophils (*p* < 0.001, OR = 2.363) and lymphocytes (*p* < 0.05, OR = 1.759), F_E_NO (*p* < 0.001, OR = 2.577), and total IgE (*p* < 0.05, OR = 1.188). All these differences were confirmed by comparing the T2-high allergic phenotype with the T2-low population. Further, T2-high allergic subjects were more often classified as never smokers (*p* < 0.01, OR = 2.107), but less as past smokers (*p* < 0.05, OR = 0.549), and had fewer blood neutrophils (*p* < 0.05, OR = 0.843). In contrast, subjects with T2-high non-allergic asthma exhibited fewer differences than the T2-low phenotype. These subjects were thinner (*p* < 0.05, OR = 0.953), had higher blood eosinophils (*p* < 0.001, OR = 2.896), lymphocytes (*p* < 0.05, OR = 1.974), and leukocyte counts (*p* < 0.05, OR = 1.177), and F_E_NO (*p* < 0.001, OR = 3.827).

Comparing the results of the two T2-high subpopulations, allergic subjects were younger (*p* < 0.001, OR = 0.952), developed asthma earlier (*p* < 0.001, OR = 0.968), and were more frequently classified as early onset (*p* < 0.001 OR = 4.047). Allergic subjects had lower BMI (*p* < 0.05, OR = 0.958) and smoke exposure (*p* < 0.01, OR = 0.979), namely were more often never smokers (*p* < 0.001, OR = 2.305) and more rarely past smokers (*p* < 0.01, OR = 0.464). In addition, they had reduced leukocyte (*p* < 0.001, OR = 0.807), blood neutrophil (*p* < 0.01, OR = 0.786), and blood eosinophil (*p* < 0.05, OR = 0.908) counts, while total IgE serum levels were increased (*p* < 0.001, OR = 1.259).

Differences in lung function parameters and treatment strategies among T2 phenotypes were reported in Table 4.

**Figure 3:**
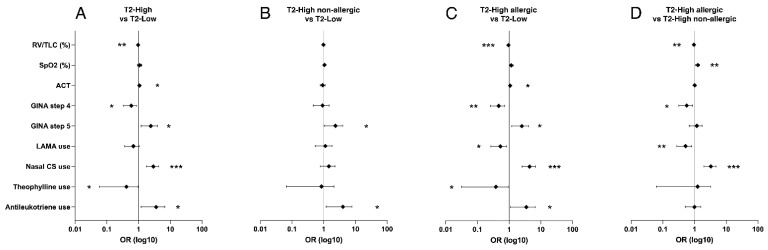
Forest plots of Odds Ratios ± 95% CI concerning lung function parameters and therapeutic strategies with significant differences between phenotypes at univariate analysis. Type 2 low is considered the reference in panels (**A**–**C**); Type 2 high non-allergic is considered the reference in panel (**D**). * = *p* < 0.05; ** = *p* < 0.01; *** = *p* < 0.001.

Evaluating the LR model (Figure 3), lung function changed only for the RV/TLC% ratio, which was lower in the T2-high phenotype (*p* < 0.01, OR = 0.962). Furthermore, compared to the T2-low, the T2-high phenotype included more likely subjects with asthma severity grade 5 (*p* < 0.05, OR = 2.168) but less with grade 4 (*p* < 0.05, OR = 0.540); however, there was greater asthma control (*p* < 0.05, OR = 1.067). Nasal CS (*p* < 0.001, OR = 2.749) and antileukotriene (*p* < 0.05, OR = 2.825) were frequently used, but there was less use of theophylline (*p* < 0.05, OR = 0.234). Results were confirmed comparing the T2-low with the T2-high allergic phenotype, which also had less LAMA use (*p* < 0.05, OR = 0.475). On the contrary, with respect to T2-low, T2-high non-allergic asthma had more frequently asthma severity grade 5 (*p* < 0.05, OR = 2.023) and used more antileukotriene drugs (*p* < 0.05, OR = 3.038), only. Comparing the two T2-high phenotypes, allergic asthmatics had lower RV/TLC% (*p* < 0.01, OR = 0.963) and higher SpO2 (*p* < 0.01, OR = 1.260). T2-high allergic phenotype rarely had severity grade 4 (*p* < 0.05, OR = 0.521), even though this phenotype, despite rarely treated with LAMA (*p* < 0.05, OR = 0.477), was the only treated with omalizumab (Table 3) and used more frequently nasal CS (*p* < 0.001, OR = 3.090).

The presence of comorbidities was reported in Table 5.

**Figure 4:**
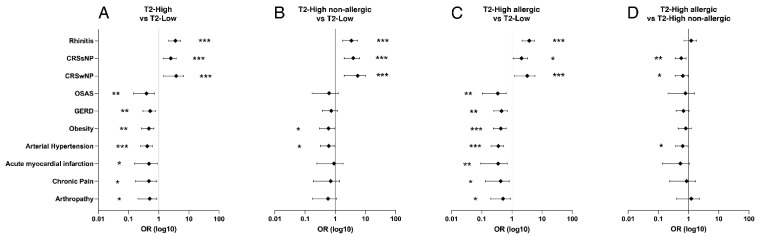
Forest plots of Odds Ratios ± 95% CI concerning comorbidity prevalence with significant differences between phenotypes at univariate analysis. Type 2 low is considered the reference in panels (**A**–**C**); Type 2 high non-allergic is considered the reference in panel (**D**). * = *p* < 0.05; ** = *p* < 0.01; *** = *p* < 0.001.

Evaluating comorbidities with LR (Figure 4), in comparison to the T2-low phenotype, T2-high subjects had a higher risk of having rhinitis (*p* < 0.001, OR = 3.358) and chronic rhinosinusitis with (*p* < 0.001, OR = 3.169) or without (*p* < 0.001, OR = 2.335) nasal polyps. Subjects with T2-high phenotype had less frequently obstructive sleep apnea syndrome (OSAS, *p* < 0.01, OR = 0.325), gastro-esophageal reflux disease (GERD, *p* < 0.01, OR = 0.485), obesity (*p* < 0.01, OR = 4.38), arterial hypertension (*p* < 0.001, OR = 0.393), and acute myocardial infarction (*p* < 0.05, OR = 0.380). Significant differences were found for chronic pain and arthropathy (*p* < 0.05), but results were not confirmed after evaluating age as a confounding factor. Results were confirmed by comparing the T2-low and the T2-high allergic cohorts. Otherwise, T2-high non-allergic subjects had a higher risk of having rhinitis (*p* < 0.001, OR = 3.092) and rhinosinusitis with (*p* < 0.001, OR = 4.451) or without (*p* < 0.001, OR = 3.528) nasal polyps, but rarely had obesity (*p* < 0.05, OR = 0.532) and arterial hypertension (*p* < 0.05, OR = 0.552) if compared to T2-low subjects. Finally, evaluating the two T2-high cohorts, allergic subjects had less rhinosinusitis with (*p* < 0.05 OR = 0.595) or without (*p* < 0.01, OR = 0.544) nasal polyps and arterial hypertension (*p* < 0.05, OR = 0.599).

## 4. Discussion

Heterogeneity of asthma has been known since the end of the 1940s when atopic and non-atopic asthma were first described. Then, the primary involvement of T_H_2-mediated inflammation in asthma pathophysiology was discovered, leading to the definition of eosinophilic and non-eosinophilic asthma. It paved the way for the consolidated evidence that it is possible to stratify asthmatics in different phenotypes. From that moment onward, a plethora of different phenotypes that consider clinical, functional, and inflammatory characteristics have been described [18,19,23,24,25]. To complicate the context further, the belonging to an asthma phenotype is not maintained over time, as described phenotypes are often characterized by overlapping traits [26]. Nevertheless, the proper identification of the specific asthma phenotype, typical of precision medicine [27], is crucial for allowing the targeted treatment tailoring (personalized medicine) [28].

However, evidence is usually derived from regulatory, experimental, or investigational studies using sophisticated parameters or strictly selected patients; that is, the obtained outcomes may be poorly representative of everyday clinical practice. The current study analyzed a large group of asthmatic outpatients in a real-world setting, using non-invasive parameters assessed in daily practice.

Despite the IgE ≥ 75 IU/mL threshold was recently used in the clustering of severe asthma [29] and IgE ≥ 100 IU/mL has been covered in the definition of T2-high allergic inflammation [19], we deliberately ruled out the serum total IgE content from our patients’ stratification. Total IgE levels have been independently associated, in non-atopic individuals, with cigarette smoking and alcohol consumption, while variable concentrations have also been detected in aging, autoimmunity, immunodeficiency (e.g., Job’s Syndrome), cancer, obesity, parasitic infection, and metabolic syndrome [30]; otherwise, allergic patients may have low total IgE levels. The IgE clinical utility has been judged modest in clinical practice [31]; hence, total IgE cannot be considered a reliable biomarker for T2-high allergic phenotyping [5,32].

The primary outcome was to demonstrate the feasibility of phenotyping asthmatics in daily clinical activity. The patients were firstly stratified in T2-low and T2-high asthma phenotypes, and then T2-high was split in T2-high allergic and T2-high non-allergic asthma. This classification reflects the main clusters of outpatients who are currently examined in an asthma clinic. Our stratification strategy identified a percentage of T2-low asthmatics (19.5%) coherent with what was recently described by Heaney and co-workers (23%) [33].

T2-low asthmatics significantly differed from T2-high, mainly concerning the comparison with the allergic phenotype, while the T2 non-allergic one has an intermediate condition.

Compared to T2-high allergic asthma, patients with T2-low asthma were older, had a later asthma onset, had more often smoking history, higher BMI, less frequently grade 5 of asthma severity, but higher RV/TLC and peripheral neutrophilia. Patients with T2-low asthma had less rhinitis, chronic rhinosinusitis [34], and more arterial hypertension, myocardial infarction, obesity, GERD and OSAS. Thereby, the T2-low asthmatic could be described as an old overweight subject with peripheral neutrophilia, heavy smoker, which led to air trapping. Obesity and asthma are closely related, and increased BMI may significantly affect asthma outcomes [35], likely due to chronic inflammation and mechanical mechanisms. T2-low asthmatics tend to have less severe asthma. Although we have no data on bronchial infiltration, it is presumable that T2-low asthmatics had neutrophilic or pauci-granulocytic inflammatory phenotypes. Consistently, pauci-granulocytic asthma is usually less severe than eosinophilic asthma [36]. T2-high allergic patients had a higher probability of being affected by more severe asthma (mainly grade 5, OR = 2.236, 95% CI 1.217–4.107, *p* < 0.05), with higher use of nasal corticosteroids (OR = 4.104, 95% CI 2.524–6.673, *p* < 0.001), and eosinophilia (OR = 2.400, 95% CI 1.826–3.153, *p* < 0.001) than patients with T2-low asthma.

These findings were consistent with previous studies that evidenced particular clinical, functional, and biological characteristics among the different phenotypes [37]. Allergic asthma is usually characterized by early onset [38], whereas non-allergic and T2-low asthma is more frequently associated with obesity and old age [39,40]. This study confirmed the association between T2-high asthma and its severity [41], that could be explained by upper airways comorbidity [42].

Here, T2-high allergic asthmatics were younger than other asthmatics consistently with the onset age and evidence that allergic patients tend to be younger than non-allergic asthmatics [43]. As reported in the literature, LAMA use was more frequent in both non-allergic phenotypes (T2-low and high) [44].

In our cohort, allergic patients had less peripheral neutrophilia than other asthmatics. Neutrophilia is characteristic of T2-low asthma [45], but it is also retrievable in the severe adult-asthma, which often enclose non-atopic patients with persistent eosinophilic inflammation [46]. Further, neutrophilia may be associated with corticosteroid resistance [45], which together with airway hyperresponsiveness correlate with an elevated level of interleukin-17 [47], whose higher levels have been demonstrated to be a marker of the frequent exacerbation asthma phenotype and severe allergic asthma [8,48]. In the present study, no difference in asthma exacerbations has been detected among the different asthma phenotypes. As the T2-high phenotypes characterized by higher asthma severity, this outcome could suggest that the T2-low phenotype may entail different endotypes. In this context, a transcriptomic and proteomic study has very recently demonstrated that T2-low asthma includes different molecular clusters associated with clinical features [49]. Hypertension and overweight were frequent comorbidities in T2-low patients. The higher frequency of patients with hypertension and elevated blood neutrophilia in our group of T2-low asthma was in line with a recent study in which patients with higher expression of T1 genes, such as interferon (IFN) family members, had a history of hypertension and high neutrophil-to-lymphocyte ratios in peripheral blood [50]. Both diseases share chronic low-grade inflammation sustained by pro-inflammatory cytokines, including IL-6 and IL-17 [51]. Hypertension skews T-cell response toward a type 1 pro-inflammatory phenotype, characterized by increased IFN-γ production and reduced T2 polarization [52]. Hyper-production of INF-γ is associated with both severe neutrophilic asthma and hypertension [53,54].

Consequently, T2-low asthma could recognize an intricate puzzle of pathogenic mechanisms characterizing the clinical features. We observed that the blood neutrophil number of T2-high non-allergic patients was significantly higher than allergic, but not than T2-low. Moreover, T2-high non-allergic patients were more likely affected by hypertension than allergic. This finding strengthens the hypothesis that the T2-high non-allergic could be considered as an intermediate phenotype. From a functional perspective, T2-low and T2-high non-allergic patients tend to have a distal airways impairment, characterized by air trapping (higher RV/TLC values), in comparison with allergic patients. This could be attributable to higher cigarette smoking. It is well-known that cigarette smoke deeply penetrates the lung up to the *lobuli* where promotes inflammation. Thus, T2-low patients are very different from T2-high allergic patients, more than non-allergic ones, considering both immunological and functional aspects. Table 6 shows a summary of the main characteristics of the different phenotypes.

The current study highlighted the typical features of both T2-low and T2-high asthma and extended to the whole mild-to-severe asthma population the recent results described by Denton et al. [29] considering five severe asthma clusters. The flip side of this study was the demonstration that many T2-high phenotypes may occur. The three biomarkers (F_E_NO, peripheral blood eosinophils, and allergen-specific IgE) identify seven combinations: four overlapping and three homogeneous. This outcome underlines the importance of using together in clinical practice, considering that biomarker positivity and measurements change over time [55], leading to sub-phenotype or even T2 phenotype switches. Nevertheless, the precise detection of biomarkers allows a personalized therapy [56].

The limitations of the present study include the cross-sectional design and the relatively high age of enrolled patients. However, a longitudinal study exploring these variables and considering repeated measurements of biomarkers over time is ongoing. Furthermore, in our T2-high population, an appreciable number of patients (*n* = 85, 16.9%) showed allergen sensitization only, without the presence of other T2 inflammation biomarkers. These latent allergic subjects could be different from the “active” T2-high population but similar to the T2-low one. Thereby, future studies are warranted to unbundle the allergic sensitization, focalizing the classification on the other T2 inflammatory biomarkers.

This real-world study demonstrates that it is possible to divide asthma patients using non-invasive parameters or parameters available in clinical practice. Although differences between groups may seem small, this type of classification still allows us to obtain populations with peculiar characteristics (Table 6) comparable to those of the more complex cluster analyses [29]. However, it should be emphasized that there are still many obscure aspects. Involvements of F_E_NO with IL-4/13 cascade and eosinophils with IL-5 cascade are established in the definition of the T2-high inflammatory process. However, the integration of alarmins (TSLP, IL-25, and IL-33) quantification to differentiate the T2-high allergic phenotype from the non-allergic it could be interesting in the future.

Similarly, the lack of knowledge and standardization for specific markers for T2-low, such as serum/sputum IL-6 and chitinase-like protein [57,58], makes this phenotype a possible set of subjects characterized solely by not being T2-high. The findings of our study suggest that sophisticated analyses would be needed to determine asthma phenotype reliably. Therefore, it would be desirable to extend the non-invasive characterization studies of T2 phenotypes by quantifying cytokine patterns at the sputum and serum level to separate better the different subgroups present in the T2-high phenotype and, hypothetically, in the T2-low phenotype.

From our basic, straightforward, but quick real-life stratification, it is possible to assert the T2-low asthma phenotype presents peculiar clinical and functional features significantly different from the T2-high allergic phenotype. In contrast, the T2-high non-allergic phenotype seems to have intermediate characteristics.

## Figures and Tables

**Table 1:** Demographic and clinical characteristics of study participants (*n* = 503). The results are expressed as mean with standard deviation or as the number of subjects with percentage.

Characteristic	Overall	Characteristic	Overall
***Demographic data***		***Asthma severity grade***	
Gender (Male/Female)	199/304	GINA step 1	53/503 (10.5%)
Age (years)	58.0 ± 15.1	GINA step 2	58/503 (11.5%)
Age at asthma onset (years)	36.0 ± 18.9	GINA step 3	150/503 (29.8%)
Early onset ◊	114/500 (22.8%)	GINA step 4	112/503 (22.3%)
Asthma duration (years)	22.4 ± 16.3	GINA step 5	130/503 (25.8%)
BMI (Kg/m^2^)	27.0 ± 5.5	ACT	20.5 ± 3.9
Never Smoker	362/503 (72.0%)	Activity limitation	4.2 ± 1.1
Past Smoker (≥10 PY)	110/503 (21.9%)	***Treatments***	
Current Smoker (≥10 PY)	31/503 (6.1%)	BCM HFA dose (µg)	329.3 ± 245.1
Pack/Year ≥10 PY (history)	30.6 ± 20.0	OCS (maintenance therapy)	25/503 (5.0%)
Exacerbations/year	1.0 ± 1.8	OCS (dependence)	73/503 (14.5%)
FE Phenotype ◊	101/465 (21.7%)	LABA use	394/503 (78.3%)
***Lung function***		LAMA use	92/503 (18.3%)
FVC (% pred.)	99.6 ± 18.6	Omalizumab	29/503 (5.8%)
FEV_1_ (% pred.)	83.8 ± 21.4	Mepolizumab	23/503 (4.6%)
FEV_1_/FVC	68.2 ± 12.3	Theophylline use	8/503 (1.6%)
∆-post-BD FEV_1_ (mL)	227.0 ± 209.7	Antileukotriene use	69/503 (13.7%)
∆-post-BD FEV_1_ (%)	11.9 ± 10.7	Nasal CS use	324/503 (64.4%)
RV (% pred.)	127.6 ± 38.3	***Physiological data***	
RV/TLC (% pred.)	44.6 ± 12.1	SpO_2_ (%)	96.6 ± 1.5
TLC (% pred.)	106.8 ± 15.1	Heart Rate (bpm)	76.8 ± 11.4
FRC (% pred.)	113.4 ± 26.1	***Phenotyping T2-high biomarkers***	
***Biological data***		Eosinophils (≥300 cells/µL)	210/503 (41.8%)
Leukocytes (cells/µL)	7282 ± 1972	F_E_NO (≥30 ppb)	236/503 (46.9%)
Neutrophils (cells/µL)	4101 ± 1482	Allergic sensitization	278/503 (55.3%)
Eosinophils (cells/µL)	312.9 ± 249.7	Polysensitization	231/278 (83.1%)
F_E_NO (ppb) †	33.6 ± 23.2	***Asthma phenotypes***	
Total IgE IU/mL † ◊	125.3 ± 125.0	Type 2 low	98/503 (19.5%)
Vitamin D (ng/mL)	26.1 ± 12.3	Type 2 high non-allergic	127/503 (25.2%)
		Type 2 high allergic	278/503 (55.3%)

† without outliers; ◊ data not available in the entire population; BMI: body mass index; PY: pack/year; FE: frequent exacerbator (≥2 exacerbation/year); FVC: forced vital capacity; FEV_1_: forced expiratory volume in the 1st second; post-BD: post-bronchodilation; RV: residual volume; TLC: total lung capacity; FRC: functional residual capacity; F_E_NO: fractional exhaled nitric oxide; GINA step: step of treatment according to the Global Initiative for Asthma; ACT: asthma control test; BCM HFA: beclomethasone hydrofluoroalkane; OCS: oral corticosteroids; OCS maintenance: requirement of at least 6 months/year of daily OCS; OCS dependence: requirement of at least 3 months/year of daily OCS or ≥3 OCS bursts in the last year; CS: corticosteroids; LABA: long-acting beta2-agonists; LAMA: long-acting muscarinic antagonists; SpO_2_: peripheral oxygen saturation.

**Table 2:** Changes in T2 biomarkers positivity in 21 T2-high severe asthma patients treated with biologics.

Pre-Biologic Phenotype	Post-Biologic Phenotype	Patients (*n*)	Biologic Drug
F+ E- S-	F+ E+ S-	1	Mepolizumab
F- E+ S-	F+ E+ S-	1	Mepolizumab
	T2 Low	1	Mepolizumab
F- E- S+	F+ E- S+	2	Omalizumab (2)
	F- E+ S+	1	Omalizumab
	F+ E+ S+	1	Omalizumab
F+ E+ S-	F+ E- S-	3	Mepolizumab (3)
F+ E- S+	F- E- S+	1	Omalizumab
F- E+ S+	F- E- S+	3	Mepolizumab (2) Omalizumab (1)
	F+ E- S+	1	Omalizumab
	F+ E+ S+	2	Omalizumab (2)
F+ E+ S+	F- E- S+	1	Mepolizumab
	F+ E- S+	3	Mepolizumab (2) Omalizumab (1)

F+ = F_E_NO ≥ 30 ppb; F- = F_E_NO < 30 ppb; E+ = blood eosinophils ≥ 300 cells/µL; E- = blood eosinophils < 300 cells/µL; S+ = presence of allergen sensitization; S- = absence of allergen sensitization. The number in brackets near the biologic drug indicates how many patients with the specific phenotype change after the treatment with that drug.

**Table 3:** Descriptive statistic of patients (*n* = 503) stratified according to asthma phenotypes. The results are expressed as mean with standard deviation or as the number of subjects with percentage.

Characteristics	Descriptive Statistic of Asthma Phenotypes			
	Type 2 Low 98 Patients	Type 2 High 405 Patients	Type 2 High Non-Allergic 127 Patients	Type 2 High Allergic 278 Patients
Age (years)	64.4 ± 13.9	56.5 ± 15.0 ***	62.9 ± 12.3	53.5 ± 15.2 ***^,§§§^
Gender (Male)	32/98 (32.6%)	167/405 (41.2%)	57/127 (44.9%)	110/278 (39.6%)
BMI	29.0 ± 6.0	26.6 ± 5.3 ***	27.4 ± 5.5	26.2 ± 5.2 ***
Age at asthma onset	44.8 ± 18.3	33.9 ± 18.5 ***	41.1 ± 17.6	30.5 ± 18.0 ***^,§§§^
Early onset	13/97 (13.4%)	101/403 (25.1%) *	13/126 (10.3%)	88/277 (31.8%) ***^,§§§^
Asthma duration (years)	19.3 ± 15.3	23.1 ± 16.3 *	21.8 ± 17.3	23.7 ± 15.8 *
Smoking				
Never Smoker	63/98 (64.3%)	299/405 (73.8%)	79/127 (62.2%)	220/278 (79.1%) **^,§§§^
Past Smoker (≥10 PY)	26/98 (26.5%)	84/405 (20.8%)	38/127 (29.9%)	46/278 (16.6%) *^,§§^
Current Smoker (≥10 PY)	9/98 (9.2%)	22/405 (5.4%)	10/127 (7.9%)	12/278 (4.3%)
Pack/Year >10 PY (history)	37.6 ± 23.3	28.3 ± 18.3 *	30.2 ± 15.8	26.8 ± 20.2 *
Vitamin D (ng/mL)	24.7 ± 12.4	26.5 ± 12.2	26.6 ± 10.5	26.4 ± 12.9
Exacerbations/years	0.7 ± 1.1	1.1 ± 1.9	1.2 ± 2.5	1.0 ± 1.6
FE phenotype	13/85 (15.3%)	88/380 (23.2%)	28/120 (23.3%)	60/260 (23.1%)
Leukocytes (cells/µL)	7150 ± 2327	7314 ± 1879	7831 ± 1868 **	7078 ± 1840 ^§§§^
Neutrophils (cells/µL)	4297 ± 1810	4054 ± 1391	4391 ± 1505	3911 ± 1318 ^§^
Eosinophils (cells/µL)	147.1 ± 64.0	353.0 ± 261.2 ***	399.9 ± 264.4 ***	331.5 ± 257.3 ***^,§^
Lymphocytes (cells/µL)	2070 ± 621	2345 ± 796 *	2385 ± 804	2326 ± 794
Total IgE (IU/mL) †◊	44.5 ± 40.0	154.4 ± 150.1 ***	67.5 ± 51.6	215.0 ± 199.4 ***^,§§§^
F_E_NO (ppb) †	15.3 ± 7.8	41.2 ± 27.0 ***	46.0 ± 25.8 ***	39.0 ± 27.3 ***^,§§^

† without outliers; ◊ data not available in the entire population; * = *p* < 0.05; ** = *p* < 0.01; *** = *p* < 0.001 vs. T2 low; ^§^ = *p* < 0.05; ^§§^ = *p* < 0.01; ^§§§^ = *p* < 0.001 vs. T2 high non-allergic; BMI: body mass index; PY: pack/year; FE: frequent exacerbator (≥2 exacerbation/year); F_E_NO: fractional exhaled nitric oxide.

**Table 4:** Lung Function and therapeutic strategies of patients (*n* = 503) stratified according to asthma phenotypes. The results are expressed as mean with standard deviation or as the number of subjects with percentage.

Characteristics	Clinical Data in the Asthma Phenotypes			
	Type 2 Low 98 Patients	Type 2 High 405 Patients	Type 2 High Non-Allergic 127 patients	Type 2 High Allergic 278 Patients
FVC (%pred.)	97.5 ± 19.6	100.2 ± 18.4	98.7 ± 19.2	100.8 ± 18.0
FEV_1_ (%pred.)	82.1 ± 21.7	84.2 ± 21.3	82.0 ± 21.7	85.2 ± 21.1
FEV_1/_FVC (%)	67.7 ± 10.9	68.4 ± 12.6	66.1 ± 10.2	69.4 ± 13.5 ^§^
∆-post-BD FEV_1_ (mL)	192.0 ± 153.7	235.5 ± 220.5	218.4 ± 166.1	243.2 ± 241.0
∆-post-BD FEV_1_ (%)	11.6 ± 9.6	12.0 ± 10.9	12.4 ± 8.8	11.8 ± 11.8
RV (%pred.)	131.9 ± 35.4	126.5 ± 39.0	128.6 ± 40.2	125.5 ± 38.5
RV/TLC (%)	49.2 ± 11.5	43.6 ± 12.0 **	47.2 ± 11.8	42.0 ± 11.7 ***^,§§^
TLC (%pred.)	106.9 ± 15.6	106.7 ± 15.0	106.4 ± 15.9	106.9 ± 14.6
FRC (%pred.)	120.6 ± 25.6	111.6 ± 26.0 *	112.0 ± 28.2	111.5 ± 24.9
SpO_2_ (%)	96.5 ± 1.5	96.7 ± 1.5	96.3 ± 1.7	96.9 ± 1.4 ^§§^
Heart Rate (bpm)	75.4 ± 10.6	77.1 ± 11.6	75.5 ± 12.2	77.8 ± 11.2
ACT	19.6 ± 4.1	20.7 ± 3.8 *	20.5 ± 3.6	20.8 ± 3.9 *
Activity limitation	4.1 ± 1.0	4.2 ± 1.1	4.2 ± 1.0	4.2 ± 1.1
Asthma severity grade				
GINA step 1	13/98 (13.3%)	40/405 (9.9%)	14/127 (11.0%)	26/278 (9.4%)
GINA step 2	8/98 (8.2%)	50/405 (12.4%)	14/127 (11.0%)	36/278 (13.0%)
GINA step 3	31/98 (31.6%)	119/405 (29.4%)	30/127 (23.6%)	89/278 (32.0%)
GINA step 4	31/98 (31.6%)	81/405 (20.0%) *	35/127 (27.6%)	46/287 (16.6%) **^,§^
GINA step 5	15/98 (15.3%)	115/405 (28.4%) *	34/127 (26.8%)	81/278 (29.1%) *
BCM HFA dose (µg)	310.2 ± 232.2	333.9 ± 248.2	366.9 ± 275.2	318.8 ± 233.8
OCS (maintenance)	6/98 (6.1%)	19/405 (4.7%)	9/127 (7.1%)	10/278 (3.6%)
OCS (dependence)	11/98 (11.2%)	62/405 (15.3%)	19/127 (15.0%)	43/278 (15.5%)
LABA use	77/98 (78.6%)	317/405 (78.3%)	99/127 (78.0%)	218/278 (78.4%)
LAMA use	24/98 (24.5%)	68/405 (16.8%)	31/127 (24.4%)	37/278 (13.3%) **^,§§^
Omalizumab use	0/98 (0.0%)	29/405 (7.2%) *	0/127 (0.0%)	29/278 (10.4%) **^,§§§^
Mepolizumab use	1/98 (1.0%)	22/405 (5.4%)	8/127 (6.3%)	14/278 (5.0%)
Nasal CS use	44/98 (44.9%)	280/405 (69.1%) ***	66/127 (52.0%)	214/278 (77.0%) ***^,§§§^
Theophylline use	4/98 (4.1%)	4/405 (1.0%)	2/127 (1.6%)	2/278 (0.7%)
Antileukotriene use	6/98 (6.1%)	63/405 (15.6%) *	21/127 (16.5%) *	42/278 (15.1%) *

* = *p* < 0.05; ** = *p* < 0.01; *** = *p* < 0.001 vs. T2 low; ^§^ = *p* < 0.05; ^§§^ = *p* < 0.01; ^§§§^ = *p* < 0.001 vs. T2 high non-allergic; FVC: forced vital capacity; FEV_1_: forced expiratory volume in the 1st second; post-BD: post-bronchodilator; RV: residual volume; TLC: total lung capacity; FRC: functional residual capacity; SpO_2_: peripheral oxygen saturation; ACT: asthma control test; GINA step: step of treatment according to the Global Initiative for Asthma; BCM HFA: beclomethasone hydrofluoroalkane; OCS: oral corticosteroids; OCS maintenance: requirement of at least 6 months/year of daily OCS; OCS dependence: requirement of at least 3 months/year of daily OCS or ≥3 OCS bursts in the last year; LABA: long-acting beta2-agonists; LAMA: long-acting muscarinic antagonists; CS: corticosteroids.

**Table 5:** Prevalence of comorbidities in patients (*n* = 503) stratified according to asthma phenotypes. The results are expressed as the number of subjects with percentage.

Characteristics	Comorbidities in Asthma Phenotypes			
	Type 2 Low 98 Patients	Type 2 High 405 Patients	Type 2 High Non-Allergic 127 Patients	Type 2 High Allergic 278 Patients
Aspirin intolerance	13/98 (13.3%)	54/405 (13.3%)	18/127 (14.2%)	36/278 (13.0%)
Rhinitis	46/98 (46.9%)	303/405 (74.8%) ***	93/127 (73.2%) ***	210/278 (75.5%) ***
CRSsNP	23/98 (23.5%)	169/405 (41.7%) ***	66/127 (52.0%) ***	103/278 (37.0%) *^,§§^
CRSwNP	8/98 (8.2%)	89/405 (22.0%) **	36/127 (28.4%) ***	53/278 (19.1%) *
Bronchiectasis	8/98 (8.2%)	38/405 (9.4%)	9/127 (7.1%)	29/278 (10.4%)
Emphysema	13/98 (13.3%)	35/405 (8.6%)	16/127 (12.6%)	19/278 (6.8%)
Pneumonia history	14/98 (14.3%)	47/405 (11.6%)	17/127 (13.4%)	30/278 (10.8%)
Recurrent bronchitis	2/98 (2.0%)	14/405 (3.5%)	5/127 (3.9%)	9/278 (3.2%)
OSAS	11/98 (11.2%)	16/405 (4.0%) **	7/127 (5.5%)	9/278 (3.2%) **
GERD	35/98 (35.7%)	86/405 (21.2%) **	34/127 (26.8%)	52/278 (18.7%) ***
Obesity	37/98 (37.8%)	85/405 (21.0%) ***	31/127 (24.4%) *	54/278 (19.4%) ***
Diabetes	8/98 (8.2%)	19/405 (4.7%)	7/127 (5.5%)	12/278 (4.3%)
Arterial Hypertension	41/98 (49.0%)	111/405 (27.4%) ***	44/127 (34.6%) *	67/278 (24.1%) ***^,§^
Acute myocardial infarction	9/98 (9.2%)	15/405 (3.7%) *	8/127 (6.3%)	7/278 (2.5%) *
Heart failure	2/98 (2.0%)	4/405 (1%)	3/127 (2.4%)	1/278 (0.4%)
Arrhythmia	8/98 (8.2%)	27/405 (6.7%)	9/127 (7.1%)	18/278 (6.5%)
Anxiety-depression	13/98 (13.3%)	56/405 (13.8%)	20/127 (15.8%)	36/278 (13.0%)
Osteoporosis	5/98 (5.1%)	32/405 (7.9%)	15/127 (11.8%)	17/278 (6.1%)
Chronic Pain	10/98 (10.2%)	17/405 (4.2%) *	7/127 (5.5%)	10/278 (3.6%) *
Arthropathy	13/98 (13.3%)	25/405 (6.2%) *	8/127 (6.3%)	17/278 (6.1%) *

* = *p* < 0.05; ** = *p* < 0.01; *** = *p* < 0.001 vs. T2 low; ^§^ = *p* < 0.05; ^§§^ = *p* < 0.01 vs. T2 high non-allergic; CRSsNP: chronic rhinosinusitis without polyps; CRSwNP: chronic rhinosinusitis with polyps; OSAS: Obstructive Sleep Apnea Syndrome; GERD: Gastro-Esophageal Reflux Disease.

**Table 6:** Summary of main phenotype characteristics.

T2 Low	T2 High Non-Allergic	T2 High Allergic
	Longer asthma duration	Younger
		Early asthma onset
		Mainly never smokers
	Higher asthma severity (GINA step 5)	Higher asthma severity (GINA step 5)
Higher RV/TLC (air trapping)		Lower RV/TLC
Lower lymphocytes counts	Higher leukocyte counts	Lower blood neutrophils counts
		Higher total IgE
Less CRSsNP	High CRSsNP	
Less CRSwNP	High CRSwNP	
Less rhinitis		
More OSAS		
More GERD		
More obesity		
More arterial hypertension		Less arterial hypertension
More acute myocardial infarction		

RV: residual volume; TLC: total lung capacity; GINA step: step of treatment according to the Global Initiative for Asthma; CRSsNP: chronic rhinosinusitis without polyps; CRSwNP: chronic rhinosinusitis with polyps; OSAS: Obstructive Sleep Apnea Syndrome; GERD: Gastro-Esophageal Reflux Disease.

## Data Availability

Data available on request from the authors.

## Supplementary Materials

The following are available online at https://www.mdpi.com/article/10.3390/biomedicines9111684/s1, Figure S1: Area-proportional Euler Diagrams of the type 2 biomarker positivity in the type 2 phenotypes, including patients before biologic treatment, Table S1: Output of the univariate analysis (*n* = 503).
