# Supplementary material for: Characterization of T2-Low and T2-High Asthma Phenotypes in Real-Life
Source: Biomedicines. 2021 Nov 13;9(11):1684. doi: 10.3390/biomedicines9111684 (PMC8615363; doi:10.3390/biomedicines9111684)
Supplement: Supplementary file 1 [file biomedicines-09-01684-s001.zip › biomedicines-1413144-supplementary.pdf]

Clinical Research Study

Characterization of T2-Low and T2-High asthma phenotypes in real-life

Fabio Luigi Massimo Ricciardolo[1], PhD, MD, Andrea Elio Sprio[1,2], PhD, Andrea Baroso[1], MD, Fabio Gallo[3], MSc, Elisa Riccardi[1], MD, Francesca Bertolini[1], PhD, Vitina Carriero[1], PhD, Elisa Arrigo[1], MSc, Giorgio Ciprandi[4], MD.

[1] Department of Clinical and Biological Sciences, University of Turin, San Luigi Gonzaga University Hospital, Turin, Italy

[2] Department of Research, ASOMI College of Sciences, Marsa, Malta

[3] Department of Health Science, University of Genoa, Genoa, Italy

[4] Allergy Clinic, Casa di Cura Villa Montallegro, Genoa, Italy

Supplementary Table S1: Output of the univariate analysis (N=503). Characteristic: variable taken into account; OR (95% CI): Odd Ratios (ExpB) with 95% Confidence Interval.

**Characteristic**	**Univariate analysis**			
	Type 2 high vs Type 2 low	*Type 2 high non-allergic Vs Type 2 low*	*Type 2 high allergic Vs Type 2 low*	*Type 2 high allergic Vs Type 2 high non-allergic*
**Age (years)**	0.960 (0.943-0.977)***	0.990 (0.970-1.012)	0.945 (0.927-0.963)###	0.952 (0.936-0.969)§§§
**Gender** (*Male)*	1.453 (0.912-2.317)	1.679 (0.971-2.906)	1.359 (0.836-2.209)	0.809 (0.529-1.237)
**BMI**	0.926 (0.890-0.924)***	0.953 (0.909-0.999)#	0.913 (0.874-0.953)###	0.958 (0.921-0.998)§
**Age at asthma onset**	0.968 (0.955-0.981)***	0.988 (0.973-1.003)	0.957 (0.944-0.971)###	0.968 (0.956-0.980)§§§
**Early onset**	2.161 (1.155-4.042)*	0.743 (0.328-1.686)	3.009 (1.592-5.686)##	4.047 (2.161-7.578)§§§
**Asthma duration (years)**	1.016 (1.001-1.031)*	1.009 (0.993-1.026)	1.019 (1.003-1.035)#	1.007 (0.994-1.021)
**Smoking**				
Never Smoker	1.567 (0.981-2.504)	0.914 (0.529-1.580)	2.107 (1.273-3.490)##	2.305 (1.454-3.654)§§§
Past Smoker	0.725 (0.436-1.205)	1.182 (0.657-2.218)	0.549 (0.317-0.951)#	0.464 (0.283-0.761)§§
Current Smoker	0.568 (0.253-1.276)	0.845 (0.330-2.168)	0.446 (0.182-1.094)	0.528 (0.222-1.256)
**Pack/Year 10PY (history)**	0.984 (0.973-0.995)**	0.996 (0.983-1.008)	0.975 (0.962-0.988)###	0.979 (0.965-0.992)§§
**BCM HFA dose (µg)**	1.000 (0.999-1.001)	1.001 (1.000-1.002)	1.000 (0.999-1.001)	0.999 (0.998-1.000)
**OCS (maintenance)**	0.755 (0.293-1.943)	1.169 (0.402-3.404)	0.572 (0.202-1.618)	0.489 (0.194-1.235)
**OCS (dependent)**	1.430 (0.722-2.830)	1.391 (0.629-3.079)	1.447 (0.714-2.933)	1.040 (0.579-1.869)
**LABA** use	0.994 (0.580-1.701)	0.964 (0.509-1.828)	1.008 (0.575-1.767)	1.045 (0.628-1.738)
**LAMA** use	0.624 (0.368-1.059)	0.996 (0.539-1.838)	0.475 (0.267-0.846)#	0.477 (0.280-0.813)§§
**Omalizumab** use	-	-	-	-
**Mepolizumab** use	5.572 (0.742-41.85)	6.521 (0.802-53.04)	5.144 (0.667-39.64)	0.789 (0.322-1.931)
**Nasal CS** use	2.749 (1.752-4.313)***	1.328 (0.783-2.253)	4.104 (2.524-6.673)###	3.090 (1.978-4.829)§§§
**Theophylline** use	0.234 (0.058-0.954)*	0.376 (0.067-2.096)	0.170 (0.031-0.945)#	0.453 (0.063-3.252)
**Antileukotriene** use	2.825 (1.185-6.732)*	3.038 (1.176-7.849)#	2.729 (1.122-6.636)#	0.898 (0.507-1.591)
**Vitamin D (ng/mL)**	1.014 (0.990-1.038)	1.015 (0.987-1043)	1.013 (0.989-1.038)	0.998 (0.978-1.019)
**Asthma severity grade**				
GINA step 1	0.717 (0.367-1.399)	0.810 (0.362-1.813)	0.675 (0.332-1.372)	0.833 (0.419-1.655)

GINA step 2	1.585 (0.725-3.461)	1.394 (0.560-3.469)	1.674 (0.749-3.737)	1.201 (0.623-2.315)

Supplementary Table S1: Output of the univariate analysis (N=503). Characteristic: variable taken into account; OR (95% CI): Odd Ratios (ExpB) with 95% Confidence Interval (continued.)

**Characteristic**	**Univariate analysis**			
	Type 2 high vs Type 2 low	*Type 2 high non-allergic Vs Type 2 low*	*Type 2 high allergic Vs Type 2 low*	*Type 2 high allergic Vs Type 2 high non-allergic*
**Asthma severity grade**				
GINA step 3	0.899 (0.558-1.448)	0.668 (0.370-1.207)	1.018 (0.621-1.669)	1.523 (0.941-2.462)
GINA step 4	0.540 (0.331-0.882)*	0.822 (0.462-1.464)	0.429 (0.252-0.728)**	0.521 (0.316-0.869)§
GINA step 5	2.168 (1.200-3.914)*	2.023 (1.029-3.976)*	2.236 (1.217-4.107)*	1.105 (0.690-1.770)
**Exacerbations/years**	1.163 (0.975-1.388)	1.208 (0.999-1.461)	1.140 (0.951-1.367)	0.945 (0.849-1.051)
**FE phenotype**	1.811 (0.964-3.401)	1.846 (0.899-3.791)	1.795 (0.937-3.440)	0.972 (0.585-1.616)
**FVC (%pred.)**	1.008 (0.996-1.020)	1.003 (0.989-1.018)	1.010 (0.997-1.022)	1.006 (0.995-1.018)
**FEV_1 (%pred.)**	1.005 (0.994-1.015)	1.000 (0.988-1.012)	1.007 (0.996-1.018)	1.007 (0.997-1.017)
**FEV_1/FVC (%)**	1.004 (0.987-1.022)	0.995 (0.974-1.015)	1.008 (0.990-1.027)	1.014 (0.997-1.031)
**-post-BD FEV_1 (mL)**	1.001 (1.000-1.003)	1.001 (0.999-1.002)	1.001 (1.000-1.003)	1.001 (0.999-1.002)
**-post-BD FEV_1 (%)**	1.004 (0.981-1.027)	1.007 (0.980-1.035)	1.002 (0.978-1.027)	0.996 (0.975-1.016)
**RV (%pred.)**	0.997 (0.991-1.003)	0.998 (0.991-1.002)	0.996 (0.989-1.002)	0.998 (0.992-1.004)
**RV/TLC (%)**	0.962 (0.940-0.984)**	0.986 (0.959-1.013)	0.950 (0.926-0.974)***	0.963 (0.943-0.984)§§
**TLC (%pred.)**	0.999 (0.982-1.015)	0.997 (0.977-1.016)	1.000 (0.983-1.017)	1.003 (0.988-1.019)
**FRC (%pred.)**	0.987 (0.975-1.000)	0.988 (0.973-1.003)	0.987 (0.974-1.000)	0.999 (0.987-1.011)
**Leukocytes** (1000 cells/µL)	1.044 (0.931-1.171)	1.177 (1.028-1.346)*	0.982 (0.874-1.103)	0.807 (0.720-0.906)§§§
**Neutrophils** (1000 cells/µL)	0.900 (0.769-1.053)	1.036 (0.865-1.241)	0.843 (0.714-0.996)*	0.786 (0.665-0.928)§§
**Eosinophils** (100 cells/µL)	2.363 (1.851-3.017)***	2.896 (2.109-3.975)***	2.400 (1.826-3.153)***	0.908 (0.839-0.982)§
**Lymphocytes** (1000 cells/µL)	1.759 (1.113-2.779)*	1.974 (1.115-3.496)*	1.671 (1.056-2.642)*	0.913 (0.656-1.271)
**Total IgE** (100 IU/mL)	1.188 (1.034-1.364)*	1.008 (0.904-1.124)	1.264 (1.087-1.471)**	1.259 (1.111-1.426)§§§
**F_ENO** (10 ppb)	2.577 (2.016-3.294)***	3.827 (2.652-5.522)***	2.394 (1.853-3.093)***	0.962 (0.902-1.026)
**ACT**	1.067 (1.010-1.126)*	1.055 (0.988-1.127)	1.072 (1.012-1.136)*	1.015 (0.961-1.073)
**Activity limitation**	1.124 (0.906-1.394)	1.113 (0.858-1.444)	1.129 (0.901-1.414)	1.014 (0.824-1.247)

**SpO2 (%)**	1.075 (0.930-1.242)	0.919 (0.773-1.092)	1.161 (0.995-1.355)	1.260 (1.094-1.451)§§
**Heart Rate (bpm)**	1.013 (0.993-1.033)	1.001 (0.978-1.025)	1.019 (0.998-1.040)	1.017 (0.999-1.036)

Supplementary Table 1: Output of the univariate analysis (N=503). Characteristic: variable taken into account; OR (95% CI): Odd Ratios (ExpB) with 95% Confidence Interval (continued.)

**Characteristic**	**Univariate analysis**			
	Type 2 high vs Type 2 low	*Type 2 high non-allergic Vs Type 2 low*	*Type 2 high allergic Vs Type 2 low*	*Type 2 high allergic Vs Type 2 high non-allergic*
**Comorbidities**				
Aspirin intolerance	1.006 (0.525-1.927)	1.080 (0.501-2.327)	0.973 (0.492-1.921)	0.901 (0.490-1.657)
Rhinitis	3.358 (2.129-5.297)***	3.092 (1.769-5.404)***	3.491 (2.156-5.652)***	1.129 (0.700-1.822)
CRSsNP	2.335 (1.406-3.878)***	3.528 (1.970-6.318)***	1.919 (1.113-3.250)*	0.544 (0.356-0.832)§§
CRSwNP	3.169 (1.481-6.778)***	4.451 (1.961-10.10)***	2.650 (1.212-5.796)***	0.595 (0.365-0.970)§
Bronchiectasis	1.165 (0.525-2.583)	0.858 (0.318-2.312)	1.310 (0.578-2.972)	1.527 (0.700-3.329)
Emphysema	0.618 (0.313-1.219)	0.948 (0.433-2.080)	0.478 (0.226-1.008)	0.504 (0.250-1.016)
Pneumonia history	0.788 (0.414-1.497)	0.927 (0.433-1.987)	0.726 (0.367-1.434)	0.783 (0.414-1.478)
Recurrent bronchitis	1.719 (0.384-7.690)	1.967 (0.373-10.36)	1.606 (0.341-7.565)	0.816 (0.268-2.487)
OSAS	0.325 (0.146-0.726)**	0.461 (0.172-1.238)	0.265 (0.106-0.660)**	0.574 (0.209-1.576)
GERD	0.485 (0.301-0.782)**	0.658 (0.372-1.164)	0.414 (0.248-0.691)**	0.629 (0.384-1.033)
Obesity	0.438 (0.273-0.703)**	0.532 (0.300-0.946)*	0.397 (0.240-0.659)***	0.747 (0.452-1.234)
Diabetes	0.554 (0.235-1.305)	0.656 (0.230-1.876)	0.508 (0.201-1.281)	0.773 (0.297-2.013)
Arterial Hypertension	0.393 (0.250-0.618)***	0.552 (0.322-0.947)*	0.331 (0.204-0.536)***	0.599 (0.379-0.946)§
Acute myocardial infarction	0.380 (0.161-0.897)*	0.665 (0.247-1.791	0.255 (0.092-0.706)**	0.384 (0.136-1.084)
Heart failure	0.479 (0.086-2.652)	1.161 (0.190-7.088)	0.173 (0.016-1.933)	0.149 (0.015-1.449)
Arrhythmia	0.804 (0.353-1.828)	0.858 (0.318-2.312)	0.779 (0.327-1.853)	0.908 (0.396-2.080)
Anxiety-depression	1.049 (0.549-2.006)	1.222 (0.575-2.598)	0.973 (0.492-1.921)	0.796 (0.440-1.439)
Osteoporosis	1.596 (0.605-4.207)	2.491 (0.873-7.110)	1.211 (0.435-3.376)	0.486 (0.235-1.008)
Chronic Pain	0.386 (0.171-0.871)*	0.513 (0.188-1.401)	0.328 (0.132-0.815)#	0.640 (0.238-1.721)
Arthropathy	0.430 (0.211-0.875)*	0.440 (0.175-1.107)	0.426 (0.199-0.913)#	0.969 (0.407-2.308)

* = P<.05; ** = P<.01; *** = P<.001 vs T2low; §= P<.05; §§= P<.01; §§§= P<.001 vs T2 high non-allergic; FE: frequent exacerbator (≥2 exacerbation/year); BCM HFA: beclomethasone hydrofluoroalkane; post-BD: post-bronchodilator.

Supplementary figure legend

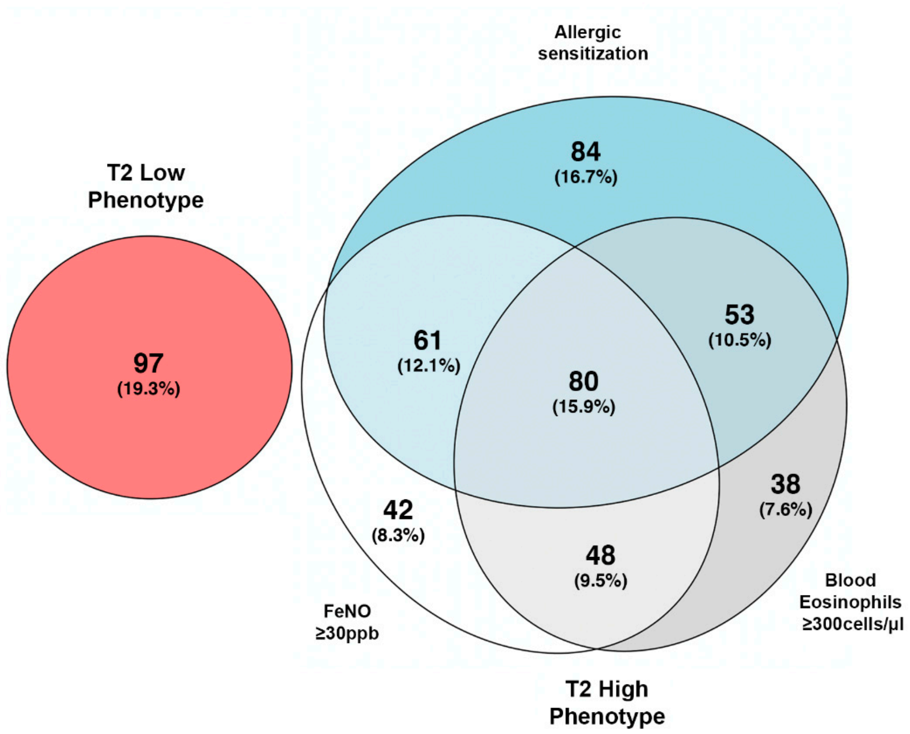

Supplementary Figure S1: Euler Diagrams of the type 2 biomarker positivity in the type 2 phenotypes including patients before biologic treatment.
